# Genome-wide identification of SIMILAR to RCD ONE (SRO) gene family in rapeseed (*Brassica napus* L.) reveals their role in drought stress response

**DOI:** 10.1080/15592324.2024.2379128

**Published:** 2024-07-14

**Authors:** Huanhuan Jiang, Yuling Zhang, Jia Li, Rongzi Tang, Fenghao Liang, Rong Tang, Yuyu Zhou, Chao Zhang

**Affiliations:** a Guizhou Oil Crops Research Institute, Guizhou Academy of Agricultural Sciences, Guiyang, Guizhou, China; b Qianxi’nan Academy of Agricultural and Forestry Sciences, Xingyi, Guizhou, China

**Keywords:** Rapeseed, similar to RCD1 (SRO), abiotic stress, gene expression, transcription factor

## Abstract

Rapeseed (*Brassica napus* L.) is an important oilseed crop widely cultivated worldwide, and drought is the main environmental factor limiting its yield enhancement and the expansion of planted areas. SIMILAR TO RCD ONE (*SRO*) is a plant-specific small gene family that plays a crucial role in plant growth, development, and responses to abiotic stresses such as drought. However, the functional role of *SROs* in rapeseed remains poorly understood. In this study, 19 *BnaSROs* were identified from the rapeseed genome, with 9, 10, 10, 18, and 20 members identified from the genomes of *Brassica rapa*, *Brassica nigra*, *Brassica oleracea*, *Brassica juncea*, and *Brassica carinata*, respectively. We then analyzed their sequence characteristics, phylogenetic relationships, gene structures, and conserved domains, and explored the collinearity relationships of the *SRO* members in *Brassica napus* and *Brassica juncea*. Next, we focused on the analysis of tissue expression and stress-responsive expression patterns of rapeseed *SRO* members and examined their expression profiles under ABA, MeJA and water-deficit drought treatments using qPCR. Transcriptome data analysis and qPCR detection indicated that *BnaSROs* exhibit multiple stress-responsive expression patterns. *BnaSRO1* and *BnaSRO11*, which are likely to function through interactions with NAC transcription factors, were screened as major drought-regulated members. Our results provide a solid foundation for functional analysis of the role of the *SRO* gene family in abiotic stress responses, especially drought stress responses, in rapeseed.

## Introduction

Rapeseed (*Brassica napus* L.) is an important oilseed crop that is grown worldwide for its edible oil and protein-rich meal.^1^ As the main source of edible vegetable oil in China, rapeseed is grown mainly in the Yangtze River valley.^2^ However, due to the uneven distribution of rainfall in the region, less rainfall in the fall and winter, seasonal droughts are severe, resulting in rapeseed seedlings often suffering from drought stress, which in turn affects their later growth and development, leading to lower yields.^3^ Drought stress has become a major factor limiting the yield improvement of rapeseed and the expansion of its cultivation area.^3,4^ Therefore, it is particularly important to identify drought response genes and analyze their molecular mechanisms to cultivate drought-resistant rapeseed cultivars.

The SIMILAR TO RCD ONE (*SRO*) gene family is a group of plant-specific genes that have been shown to play important roles in plant responses to various environmental stresses, including drought stress.^5–7^ *Arabidopsis* RADICAL-INDUCED CELL DEATH1 (*RCD1*) was the first *SRO* member to be identified.^8^ This family was subsequently identified based on the conserved domains of Poly ADP-ribose polymerase (PARP, PF00644) and RCD-SRO-TAF4 (RST, PF12174) at the C-terminus. In addition, some SRO proteins also had a WWE domain (PF02825) at the N-terminus.^5^ Among these three structural domains, PARP is mainly responsible for various biological functions, while RST and WWE are mainly involved in protein interactions.^8,9^ In *Arabidopsis*, there are six members (*AtRCD1* and *AtSRO1-5*) of this family.^9^ Among them, *AtRCD1* and *AtSRO1* share the same structural domains and exhibit partially redundant functions.^8,10^ Both are involved in the oxidative stress response in plants and respond to a variety of stress-related hormones.^8^ Double mutations strongly affect the growth and development of plants.^10,11^ Functional mutations in *AtRCD1* increase plant sensitivity to salt and osmotic stress.^10^ In addition, AtRCD1 elevates oxidative stress and drought tolerance in plants by interacting with SOS1 and DREB2A, respectively, in *Arabidopsis*.^12,13^

As an increasing number of plant genomes have been sequenced, the *SRO* gene family has been consecutively identified in different plants, such as rice,^6^ maize,^14^ wheat,^15^ tomato,^16^ cotton,^17^ potato,^18^ sesame,^19^ banana,^20^ tea plant^21^ and poplar,^22^ and the understanding of their functions is expanding. In rice, the five *SRO* genes can respond to a multitude of abiotic stresses,^23^ in which *OsSRO1c* is directly regulated by the transcription factor gene *SNAC1* to enhance rice drought and oxidative stress tolerance.^6^ In wheat, 30 *SRO* members showed different response patterns to biological and abiotic stresses,^15^ and the *TaSRO1* gene was able to enhance seedling growth and abiotic stress resistance by regulating redox homeostasis and maintaining genomic integrity.^24^ In maize, all six *SRO1* genes are inducible by a variety of abiotic stressors,^14^ and overexpression of *ZmSRO1e* in maize and *Arabidopsis* increases the sensitivity of transgenic lines to abiotic stress. Additionally, *ZmSRO1e* responds to abiotic stress by interfering with the formation of the MBW complex and inhibiting anthocyanin synthesis.^25^ In banana, the *MaSRO4* gene regulates downstream signaling pathways by interacting with MaNAC6 and MaMYB4 in a positive response to biotic and abiotic stressors.^20^ However, currently, only one *SRO* gene has been reported in rapeseed, and its study is limited to sequence analysis.^26^ The number of members of the *SRO* gene family in rapeseed and their response to adverse stressors, especially drought stress, are unknown.

In this study, we first performed genome-wide identification of the *SRO* gene family in rapeseed and five other genotypes of *Brassica* crops (*Brassica rapa*, *Brassica nigra*, *Brassica oleracea*, *Brassica juncea*, and *Brassica carinata*). We then analyzed their sequence characteristics, phylogenetic relationships, gene structures, and conserved domains, and explored the collinearity relationships of the *SRO* members in *Brassica napus* and *Brassica juncea*. Next, we focused on the analysis of tissue expression and stress-responsive expression patterns of rapeseed *SRO* members and examined their expression profiles under ABA, MeJA and water-deficit drought treatments using qPCR. Finally, we screened two potential drought-responsive members and constructed their initial transcriptional regulatory networks. Our results lay the foundation for analyzing the function and stress tolerance regulation of *SROs* and provide theoretical references for the mining of drought-tolerance genes and drought-tolerance breeding in rapeseed.

## Materials and methods

### Identification of SRO genes in six *Brassica* species

The genome sequences of *Brassica rapa* ssp.*pekinensis* (Chiifu.v4), *Brassica nigra* (Ni100 LR v2.0), *Brassica oleracea* var.*italica* (HDEM.v0) and *Brassica napus* ssp.*oleifera* (ZS11.v0) were downloaded from the BnIR database^27^ (https://yanglab.hzau.edu.cn/BnIR). The genome sequences of *Brassica juncea* (Braju_AU213_V1.0) and *Brassica carinata* (zd-1) were downloaded from the BRAD database^28^ (http://www.brassicadb.cn/#/) and BIO2DB database (http://brassicadb.bio2db.com/),^29^ respectively. The amino acid sequences of the SRO genes of *Arabidopsis thaliana* (AtRCD1, AtSRO1–5) were acquired from the TAIR database (https://www.arabidopsis.org/) and used as queries for BLASTp searches against the local annotated protein database of the above six *Brassica* genomes with an E-value cutoff <1e^−5^. The candidate protein sequences were first filtered out if they were less than 100 amino acids (AAs) in length and then further identified using Pfam (https://www.ebi.ac.uk/Tools/pfa/pfamscan/), CDD (https://www.ncbi.nlm.nih.gov/Structure/bwrpsb/bwrpsb.cgi) and SMART (https://smart.embl.de/). The members containing at least one PARP domain (PF00644) or RST domain (PF12174) were reserved for subsequent analysis.

The GFF annotation files of the identified *SRO* genes were used to visualize their genomic distribution by TBtools software.^30^ Then, they were renamed separately according to their distribution order on different genomic chromosomes. The isoelectric points (pIs), molecular weights (MWs) and grand average hydropathicity (GRAVY) values of the SRO proteins were predicted using an online tool on the ExPASy server (https://web.expasy.org/protparam/). The subcellular localization of each protein was predicted using WoLF PSORT (https://wolfpsort.hgc.jp/).^31^

### Phylogenetic, gene structure, and conserved domain analyses

The complete amino acid sequences of the different SRO genes were used for phylogenetic analysis. Sequence alignment was first performed using ClustalX software, and then phylogenetic tree construction based on the neighbor-joining (NJ) method with 1000 bootstrap replicates was carried out using MEGA6 software.^32^ The phylogenetic tree was visualized using the iTOL online tool (https://itol.embl.de/).^33^ The exon-intron structures of each *SRO* gene were analyzed and visualized based on their GFF annotation files using the TBtools software. The conserved motifs of each SRO protein were predicted using the Multiple Em for Motif Elicitation (MEME) online tool (http://meme-suite.org/tools/meme)^34^ based on the default parameters, and the maximum motif number was set to 15. The conserved motifs were subsequently visualized with TBtools software.

### Genome collinearity analysis and Ka and Ks calculations

Genome-wide collinearity analyses of *Brassica napus* (Bna), *Brassica juncea* (Bju), and Bna and Bju were performed using the “One Step MCScanX” plugin of TBtools software and visualized by the “Advanced Circos” or “Dual Systeny Plot for MCScanX” plugins. The nonsynonymous substitution rate (Ka) and synonymous substitution rate (Ks) were estimated for SRO gene pairs collinear between Bna and Bju using TBtools software. Ka/Ks < 1 indicates purifying selection or negative selection, and Ka/Ks > 1 indicates positive selection.^35^

### *Cis*-acting element prediction and expression pattern analysis

The 2 kb upstream sequence of the start codon of each gene was considered the promoter region, and the presumed *cis*-regulatory elements of each promoter were analyzed using the PlantCARE online tool (http://bioinformatics.psb.ugent.be/webtools/plantcare/html/).^36^ Data on the expression of *BnaSROs* in different tissues of rapeseed and in rapeseed leaves and roots in response to salt, drought, freezing, cold, heat, osmotic stress, and ABA and JA treatments were downloaded from the BnIR database. The visualized heatmaps were drawn using TBtools software.

### Plant materials and treatments

In this study, the ‘Zhongshuang 11’ rapeseed cultivar was used as materials. Seedlings were planted in nutrient soil in the greenhouse of the Oil Crops Research Institute of Guizhou Academy of Agricultural Sciences under 16 h of light (22°C) and 8 h of darkness (20°C). The seedlings were treated when they had four complete leaves. For the ABA and MeJA treatments, final concentrations of 100 μM ABA and 50 μM MeJA, respectively, were prepared and dissolved in sterile water containing 0.05% anhydrous ethanol and 0.01% Tween 20. A uniform spray was applied to the front and back leaves of the seedlings, and samples were taken at 0, 0.5, 1, 2, 4, and 8 h after spraying. For the drought treatment, 2 d after the watering of seedlings stopped under normal cultivation management was used as the starting point (0 d) for the drought treatment. Samples were taken at 0, 1, 3, 5, 7, and 10 days after drought treatment. In all the treatments, the top two young leaves of the seedlings were sampled, and the samples were snap-frozen in liquid nitrogen and stored in a −80 refrigerator for later use. Three biological replicates were used for each treatment.

### RNA extraction, reverse transcription and quantitative real-time PCR (qPCR) analysis

For RNA extraction, leaves were first ground to a powder under liquid nitrogen, and then total RNA was extracted using a TaKaRa MiniBEST Plant RNA Extraction Kit (TaKaRa, Japan) according to the manufacturer’s instructions. RNA quality and concentration were detected using 1.5% agarose gel electrophoresis and a nucleic acid concentration meter (ND-1000 spectrophotometer, Thermo Fisher, USA), followed by reverse transcription using the EasyScript All-in-One First-Strand cDNA Synthesis SuperMix for qPCR (One-Step gDNA Removal) Kit (TIANGEN, China). The TB Green Premix Ex Taq™ II (TaKaRa, Japan) was used for qPCR. The 10 µL reaction system contained 5 µL of TB Green Premix Ex Taq, 0.3 µL of forward and reverse primers, 1 µL of template, and 3.4 µL of RNA-free sterile water. The qPCR was performed using a Bio-Rad CFX96 instrument (Bio-Rad, USA) as previously described.^37^ The *BnaActin* gene was used as an internal control. The analysis of each sample was repeated three times, and the relative expression levels of each gene were calculated using the 2^−ΔΔCT^ method.^38^ The primers used for qPCR analysis are listed in Table S1.

## Results

### Identification of the SRO genes in rapeseed and five other *Brassica* species

In this study, a total of 19 *SRO* genes were identified from the ‘Zhongshuang 11’ rapeseed genome (AACC, *BnaSRO1-BnaSRO19*). In addition, 9, 10, 10, 18, and 20 *SRO* genes were also identified from *Brassica rapa* (AA, *BraSRO1-BraSRO9*), *Brassica nigra* (BB, *BniSRO1-BniSRO10*), *Brassica oleracea* (CC, *BolSRO1-BolSRO10*), *Brassica juncea* (AABB, *BjuSRO1-BjuSRO18*), and *Brassica carinata* (BBCC, *BcaSRO1-BcaSRO20*), respectively. The genes showed a variable distribution across the genomes of these six *Brassica* crops (Table S2). Further analysis of these SROs for protein physicochemical properties revealed that their amino acid numbers ranged from 291(BcaSRO14 and BcaSRO15) to 584 (BolSRO7), their molecular weights ranged from 31.94 kDa (BcaSRO15) to 64.39 kDa (BolSRO7), their isoelectric points ranged from 5.12 (BnaSRO19) to 9.33 (BniSRO6), and their GRAVY values ranged from −0.22 to −0.5. Subcellular localization prediction analyses showed that most of these proteins may be localized in the nucleus, while the rest may be localized in the cytoplasm, chloroplasts, or peroxisomes.

### Phylogenetic analysis of SROs in angiosperms

**Figure 1.**
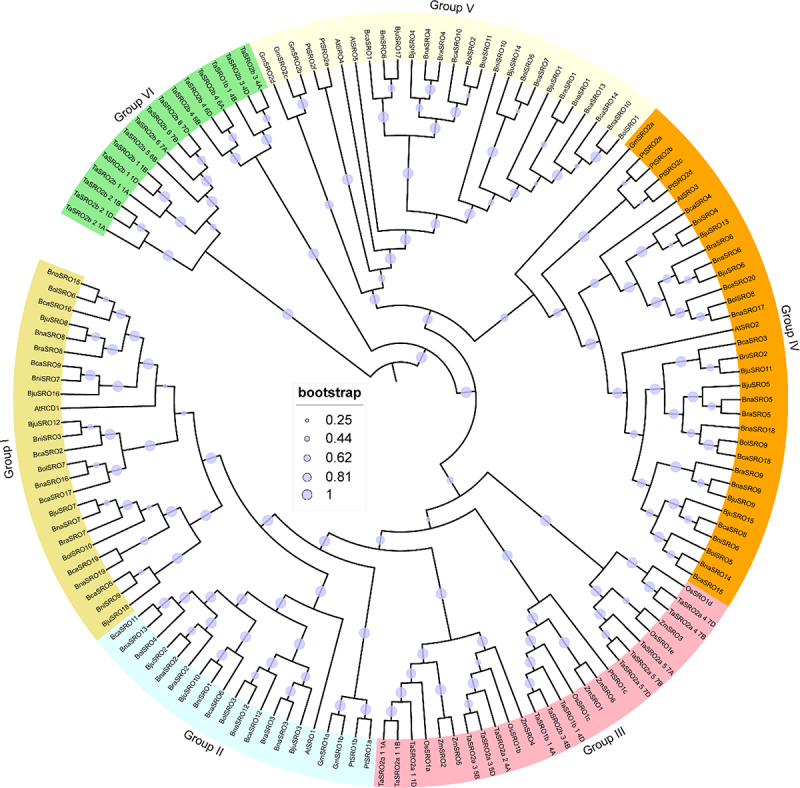
Phylogenetic analysis of the SRO genes in different plants. The accession numbers of the other plant SRO proteins used for evolutionary tree construction are listed in Table S3.

To investigate the evolutionary relationships of the SROs of these six *Brassica* crops, a phylogenetic tree was constructed with them using SROs from *Arabidopsis thaliana* (At), rice (*Oryza sativa*, Os), maize (*Zea mays*, Zm), soybean (*Glycine max*, Gm), wheat (*Triticum aestivum*, Ta), and *Populus trichocarpa* (Pt). As shown in Figure 1, these SROs were categorized into six subgroups (Group I-Group VI). The SROs of *Brassica* crops were distributed in Groups I, II, IV, and V, which correspond to the AtRCD1, AtSRO1, AtSRO2 and AtSRO3, and AtSRO4 and AtSRO5 members of *Arabidopsis*, respectively, and members of these subgroups were all derived from dicots. All the members of Group VI were from wheat, and all the members of Group III except PtSRO1c were monocotyledons.

### Gene structure and conserved motif analysis of SROs in *Brassica*

**Figure 2.**
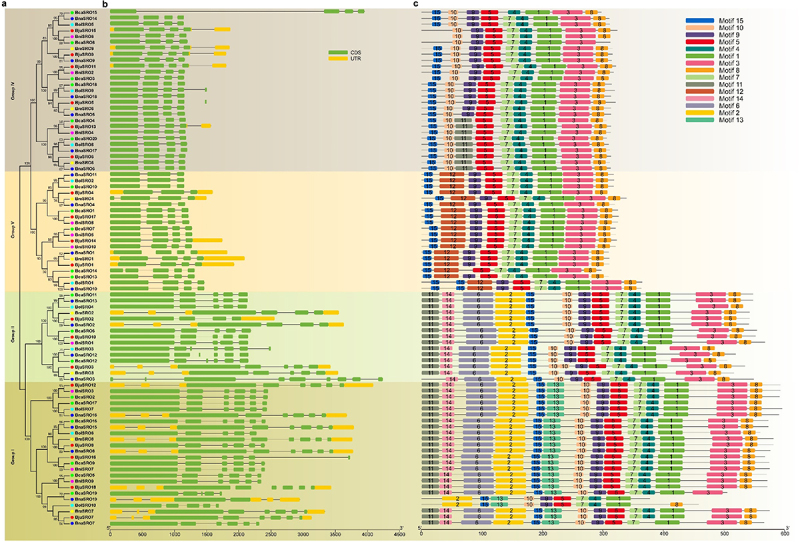
Phylogenetic tree (a), gene structure (b) and conserved motifs (c) of SROs in *Brassica*. The sequence information of these 15 conserved motifs is shown in Figure S1.

The phylogenetic tree reconstruction of only the 86 SROs from these six *Brassica* species also supported their classification into four subgroups (Figure 2(a)). Members within the same clade exhibited similar intron and exon structures (Figure 2(b)). Among them, members of Group IV possess 3–4 introns, members of Group V have 2–3 introns, members of Group II contain no less than 4 introns except *BraSRO2*, *BjuSRO2* and *BnaSRO2*, and members of Group I possess 4–5 introns. In addition, except for *BnaSRO3* in Group II, the length of the first exon of all members of Groups I and II was longer than that of Groups IV and V. Based on the amino acid sequences of these 86 SROs, a total of 15 conserved structural domains were identified (Figure 2(c)). The composition and distribution of the conserved structural domains also showed a high degree of similarity within subgroups. Among these 15 conserved motifs, only Motif 1 and Motif 5 were present in all the members, Motif 12 was present only in the members of Group V, Motif 13 was present only in the members of Group I, and Motif 2, Motif 6 and Motif 14 were found only in the members of Groups I and II.

### Collinearity analysis of SROs in *Brassica napus* and *Brassica juncea*

**Figure 3.**
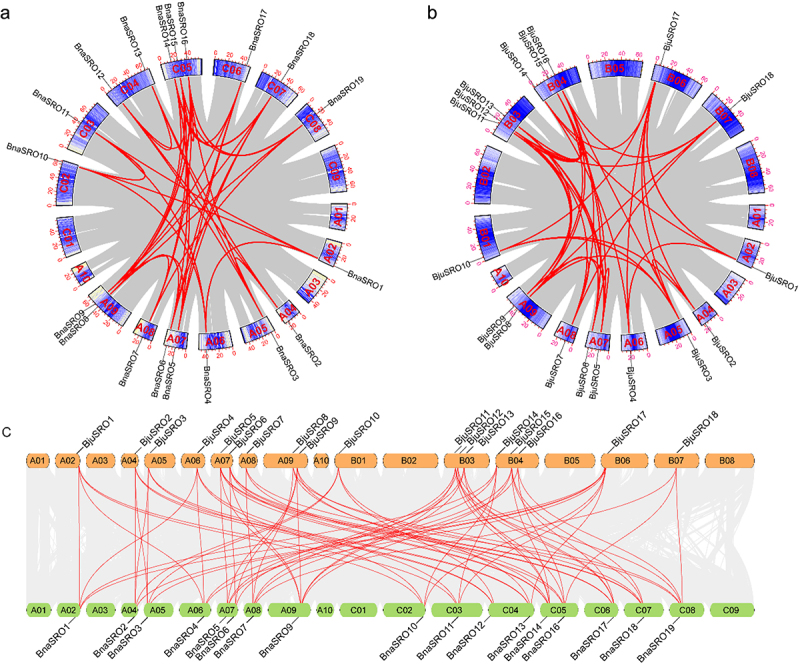
Collinearity of the *SRO* gene pairs. (a) Collinearity analysis of the *SRO* gene family in *Brassica napus*. (b) Collinearity analysis of the *SRO* gene family in *Brassica juncea*. (c) collinearity analysis of *SRO* genes between *Brassica napus* and *Brassica juncea*. The identified *SRO* gene pairs are connected by red lines.

To further characterize the evolution of *SROs* in rapeseed, collinearity analyses of *SRO* genes in *Brassica napus* and *Brassica juncea* were performed. As shown in Figure 3, a total of 37 pairs of segmentally duplicated genes were found in the *Brassica napus* genome (Figure 3(a)), and 37 collinear gene pairs were also identified in the *Brassica juncea* genome (Figure 3(b)). A total of 72 syntenic gene pairs were identified between the *Brassica napus* and *Brassica juncea* genomes (Figure 3(c)). A large number of multigene colineages existed both within the genome and between these two genomes. For example, *BnaSRO1*, *BnaSRO6*, and *BnaSRO7* have 3, 4, and 4 collinearity genes, respectively, in the *Brassica napus* genome. The genes *BjuSRO2*, *BjuSRO5*, and *BjuSRO7* had 4, 5, and 4 collinearity genes, respectively, in the *Brassica juncea* genome. *BnaSRO1* is simultaneously collinear with the *BjuSRO1*, *BjuSRO4*, *BjuSRO14*, and *BjuSRO17* genes. However, the *BnaSRO8* gene has no collinear genes in the *Brassica juncea* genome. We further analyzed the Ka/Ks values of the 72 collinear gene pairs between the *Brassica napus* and *Brassica juncea* genomes, and all of the Ka/Ks ratios were less than 1, indicating that they all underwent purifying selection during evolution (Table S4).

### Promoter analysis of *BnaSROs*

**Figure 4.**
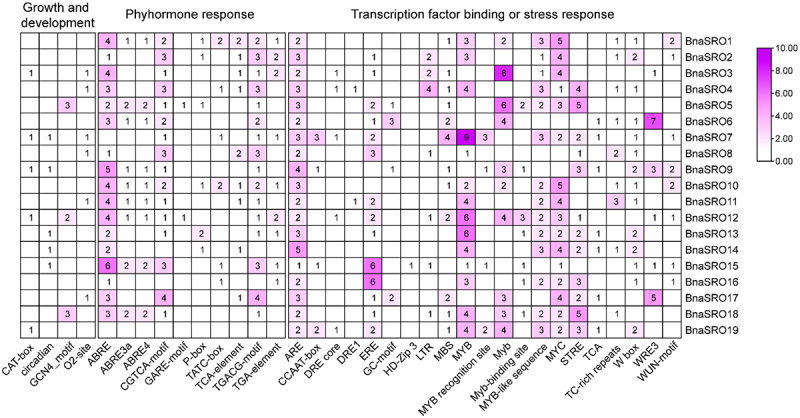
Statistics on the number of *cis*-acting elements in the promoter region of the *BnaSRO* genes. The elements were categorized into three groups based on their functions: plant growth and development, phytohormone response, and transcription factor binding or stress response. Heatmaps were drawn based on the number of different elements using TBtools software.

To explore the potential functions of rapeseed *SRO* genes, the *cis*-acting elements in their promoter regions were predicted and analyzed. As shown in Figure 4, these elements can be classified into three categories, namely, growth and development-related, hormone-responsive, and transcription factor binding or stress-responsive. For these hormone response-related elements, ABA (ABRE) and MeJA response-related elements (TGACG-motif and CGTCA-motif) were the most abundant and most widely distributed among the 19 SRO members in rapeseed, suggesting that the function of *BnaSROs* may be regulated by ABA and MeJA. Transcription factor binding or stress-responsive elements showed diverse distribution characteristics among different members of the rapeseed *SRO* family. Among them, three classes of transcription factors, *MYB*, *MYC*, and *WRKY*, had the greatest number of binding elements associated with them, indicating that the same *SRO* gene may be regulated by multiple transcription factors at the same time. In addition, low temperature-responsive element (LTR), drought-responsive element (MBS), defense and stress-responsive element (TC rich repeats), and wound-responsive element (WRE3 and WUN-motif) also exhibited variable distributions among the different members, which suggests diverse stress responses in the rapeseed SRO family along with functional differences among these members.

### Expression pattern analysis of *BnaSROs* based on transcriptome data

**Figure 5.**
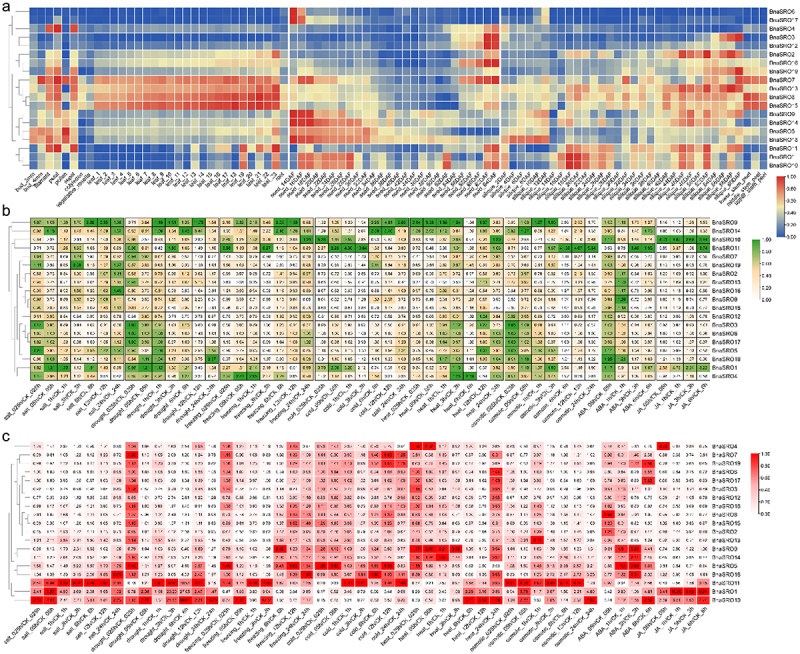
The expression patterns of *BnaSROs* in rapeseed based on transcriptome data. (a) The expression patterns of *BnaSROs* in different tissues or developmental periods of rapeseed. (b) The expression patterns of *BnaSROs* under salt, drought, freezing, cold, heat, and osmotic stresses, as well as under ABA and JA treatments, in rapeseed leaves and roots (c).

To further investigate their functional diversity, we analyzed the expression patterns of individual members of the rapeseed *SRO* family in different tissues of rapeseed and under various adverse conditions and hormonal treatments using transcriptome data from public databases (Figure 5). Expression patterns based on 91 different tissues or developmental periods showed the diversity of tissue expression of these 19 members, with different members exhibiting tissue-specific expression patterns (Figure 5(a)). Among these genes, *BnaSRO7*, *BnaSRO8, BnaSRO13*, and *BnaSRO15* presented relatively high expression levels in leaf tissues, and *BnaSRO5*, *BnaSRO9*, *BnaSRO14*, and *BnaSRO18* were highly expressed in early developing seeds. Moreover, *BnaSRO4* was specifically expressed in filaments, petals and sepals; *BnaSRO6* and *BnaSRO17* were exclusively expressed in seed_14DAF and seed_16DAF; *BnaSRO3* and *BnaSRO12* were mainly expressed in seed_62DAF and seed_64DAF; and *BnaSRO19* was predominantly expressed in siliques in the late flowering period.

For stress and hormone responses, we analyzed the expression patterns of these 19 members under salt, drought, freezing, cold, heat, and osmotic stresses, as well as under ABA and JA treatments in rapeseed leaves (Figure 5(b)) and roots (Figure 5(c)), respectively. They also showed a variety of expression patterns in response to different stresses, and the degree of response in leaf tissue and root tissue was not consistent. In terms of expression in leaf tissues (Figure 5(b)), *BnaSRO11* showed the most dramatic response to osmotic stress, and its expression level was up-regulated more than 40-fold compared with that of the control at 6 h after treatment and remained up-regulated nearly 10-fold at 24 h after treatment. In addition, its expression was also substantially induced by freezing and cold treatments as well as ABA and JA treatments. The expression pattern of *BnaSRO10* was basically the same as that of *BnaSRO11*, but to a lesser degree. The expression of *BnaSRO9* and *BnaSRO14* was induced by almost all of the above treatments, and both were induced to the greatest extent at 12 h after the drought and cold treatments. Both *BnaSRO10* and *BnaSRO1* were significantly induced in response to the JA treatments with a consistent trend. In terms of expression in root tissues (Figure 5(c)), *BnaSRO1*, *BnaSRO10* and *BnaSRO11* were significantly induced by salt, drought, freezing, heat, osmotic and JA treatments, especially drought treatment. In addition, in both leaf and root tissues, *BnaSRO9* and *BnaSRO14* as well as *BnaSRO5* and *BnaSRO18*, showed similar expression patterns in response to stress, suggesting redundancy in their functions.

### Expression profiles of *BnaSROs* under ABA, MeJA and drought treatments

**Figure 6.**
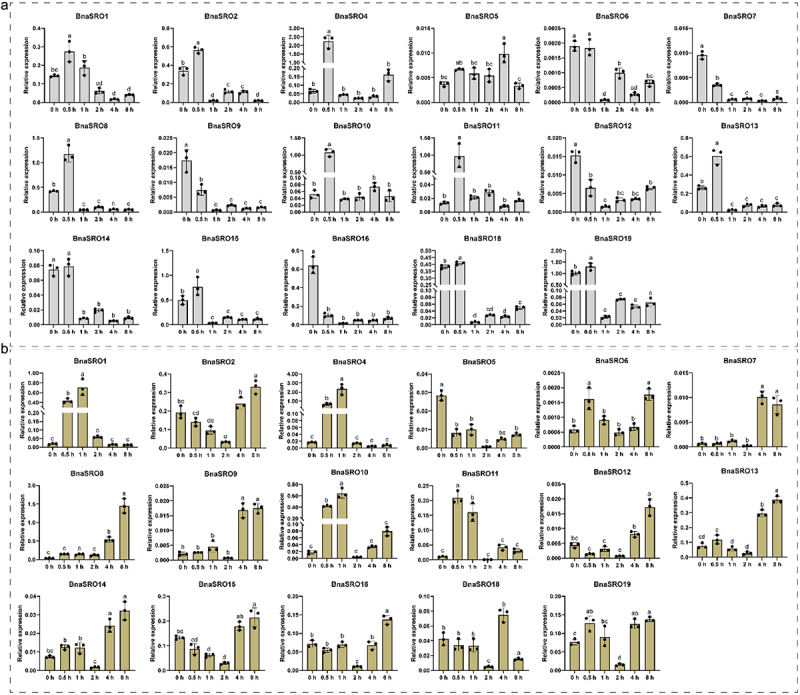
The relative expression levels of the *BnaSROs* in rapeseed leaves at different time points after exogenous ABA (a) and MeJA (b) treatments. The expression levels were calculated based on the 2^−ΔCT^ method relative to the internal reference gene. The bars represent the mean ± SD (*n* = 3). The different letters indicate significant differences at *p* < 0.05 according to Duncan’s test.

**Figure 7.**
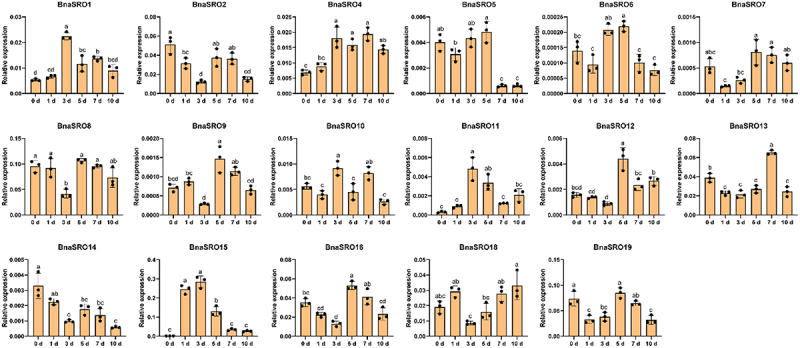
The relative expression levels of *BnaSROs* in rapeseed leaves after a different number of days of water deficit drought treatment. The expression levels were calculated based on the 2^−ΔCT^ method relative to the internal reference gene. The bars represent the mean ± SD (*n* = 3). The different letters indicate significant differences at *p* < 0.05 according to Duncan’s test.

To further clarify the potential biological functions of rapeseed *SRO* family members, we analyzed their expression under ABA, MeJA and drought treatments using qPCR. As shown in Figure 6, since the expression levels of *BnaSRO3* and *BnaSRO17* were too low to be accurately detected by qPCR, we analyzed the expression characteristics of the remaining 17 members under ABA (Figure 6(a)) and MeJA (Figure 6(b)) treatments. With the exception of *BnaSRO6*, *BnaSRO7, BnaSRO9, BnaSRO12* and *BnaSRO16*, the expression of the remaining 12 members increased at 0.5 h after ABA treatment. However, all members were down-regulated to varying degrees afterward, except for *BnaSRO4, BnaSRO5, BnaSRO10* and *BnaSRO11*, which recovered to the pre-treatment level, the expression levels of the remaining members were lower than the pre-treatment level. In the MeJA treatment group, all members exhibited distinct expression characteristics compared with those in the ABA treatment group. Among them, except for *BnaSRO5*, the expression of the other members increased to different degrees after treatment, but the time of induction was inconsistent. *BnaSRO1, BnaSRO10* and *BnaSRO11* were up-regulated to significant levels at 0.5 and 1 h after treatment, while *BnaSRO7, BnaSRO8, BnaSRO9, BnaSRO13* and *BnaSRO14* reached significant levels at 4 and 8 h after treatment. Under drought stress, as shown in Figure 7, more than half of the members were induced by drought stress. Among these genes, *BnaSRO1, BnaSRO10*, *BnaSRO11* and *BnaSRO15* were induced to the greatest extent at 3 d after treatment, *BnaSRO6, BnaSRO9*, *BnaSRO12* and *BnaSRO16* peaked at 5 d after treatment, and *BnaSRO4* and *BnaSRO13* were induced to the greatest degree at 7 d after treatment. Notably, *BnaSRO1*, *BnaSRO4* and *BnaSRO11* were still induced 10 d after drought, suggesting that they have specific functions in the drought stress response in rapeseed.

### Co-expression network construction of *BnaSRO1* and *BnaSRO11*

**Figure 8.**
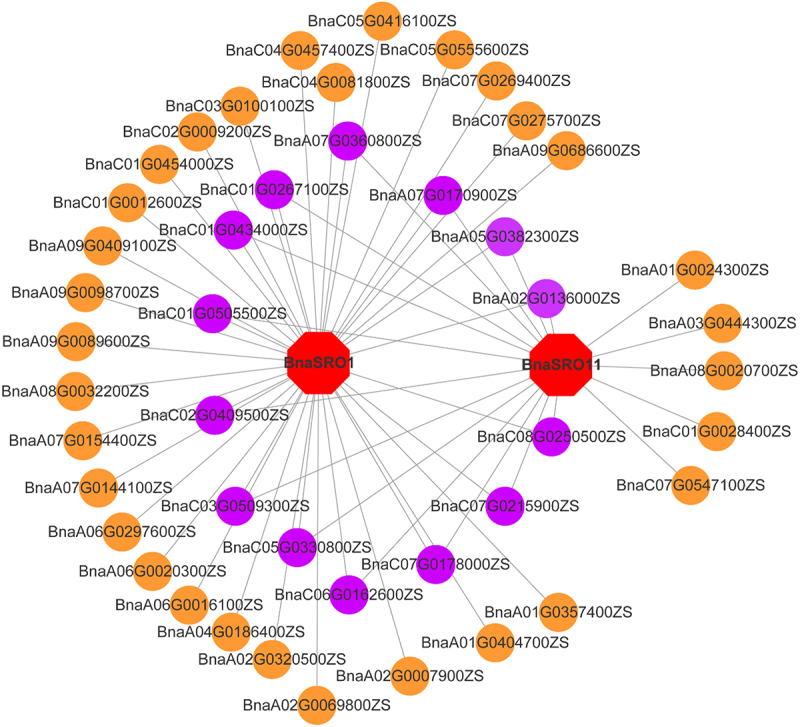
Transcriptional regulatory network of the *BnaSRO1* and *BnaSRO11* genes. The transcription factor genes are indicated by circles, and those in purple indicate transcription factors that have regulatory relationships with both *BnaSRO1* and *BnaSRO11*. The homologous gene information for these transcription factor genes in *Arabidopsis* is listed in Table S5.

Synthesizing the transcriptome data and qPCR results, we initially screened *BnaSRO1* and *BnaSRO11* as functionally important members of the rapeseed SRO family for subsequent functional studies. We constructed transcription factor (TF) regulatory networks of these two members based on public transcriptome data. As shown in Figure 8, a total of 45 TFs were identified, 14 of which may regulate both *BnaSRO1* and *BnaSRO11*. The functional annotation of these TFs showed that most were NAC family members (Table S5), implying that the functions of *BnaSRO1* and *BnaSRO11* are likely regulated by NAC transcription factors.

## Discussion

Plants have developed complex mechanisms to cope with and adapt to a variety of environmental stressors during long-term evolution, and plant-specific genes play an indispensable role in this process.^39,40^ *SROs*, as plant-specific genes, have been consistently identified in various plants, indicating their the conserved distribution in plants. They are receiving sustained attention for their special roles in plant responses to abiotic and oxidative stressors.^5,16,22^ However, studies on their molecular functions and regulatory mechanisms are currently limited to partial members of model plants such as *Arabidopsis*,^8,12^ rice,^6,23^ and maize.^25^ Nothing is known about their function in rapeseed. In this study, we systematically identified *SRO* family members in six *Brassica* crops, including rapeseed, and analyzed their sequence characteristics, physicochemical properties, structural features, and evolutionary relationships. Unlike the common diploid crops, which have 5–6 members of the *SRO* family,^6,14,16^ the tetraploid *Brassica napus*, *Brassica juncea*, and *Brassica carinata* have 19, 18, and 20 members, respectively, while *Brassica rapa*, *Brassica nigra*, and *Brassica oleracea*, which are also diploids, have 9, 10, and 10 members, respectively. This finding predicts the expansion of *SRO* family members in *Brassica* crops. This phenomenon was also detected in diploid tea plants, which possess 9 members.^21^ Given that *Brassica napus* and *Brassica juncea* are co-cultivated in southwestern China, we focused on comparing the collinearity characteristics of *SRO* genes in these two crops. A large number of segmental duplication gene pairs were identified both within and between these two genomes, and multiple duplicated genes also existed for the same gene. This explains the expansion of *SRO* family members in *Brassica*. Interestingly, *BnaSRO8* is the only member that does not have a collinear gene in *Brassica juncea*, suggesting that it is unique to *Brassica napus*. Phylogenetic analyses with different plant SROs revealed that the 86 SRO members of the abovementioned six *Brassica* species could be categorized into four subgroups, and members within the same subgroup presented similar gene structures and conserved motif compositions. This phenomenon has also been found in other plant *SROs* studies as well as in other gene family identifications,^18,19,41,42^ further demonstrating their close evolutionary relationship. In addition, our evolutionary tree results showed that, with the exception of individual members, the SROs of monocotyledonous and dicotyledonous plants were in separate subgroups. It has been suggested that the genome evolution of *SROs* accompanied the divergence of monocotyledons and dicotyledons.^15^

According to the results of the *cis*-acting elements analysis of the promoters of *BnaSROs*, each member has a large number of stress-responsive and hormone-responsive related elements, indicating that, similar to *SROs* in other plants, *BnaSROs* could be involved in the regulation of the stress response in rapeseed. Among these hormone response elements, the ABA and MeJA response elements were the most abundant. The phytohormone ABA plays a crucial role in drought responses,^43^ and the *MdRCD1* gene in apple plants positively regulates drought tolerance through the ABA signaling pathway.^44^ This indicates that members of the *BnaSRO* family regulate the response to drought stress in rapeseed. In addition, it has been demonstrated that *SROs* are also regulated by TFs during the drought stress response in plants,^9,23^ and we also identified a large number of TF binding elements on the promoters of *BnaSROs*, suggesting that this regulatory role is conserved among plants. Meanwhile, that the composition of the *cis*-acting elements in the promoter regions of the different *BnaSRO* family members varied, indicating the diversity of their functions. This functional diversity was further validated by transcription patterns. Based on public transcriptome data, we found that *BnaSROs* responded to a variety of stresses, including salt, drought, freezing, cold, heat, and osmotic stressors, to varying degrees. Under the same conditions, the response in the root tissue was greater than that in the leaves. This suggests that, similar to *SROs* in other plants,^19,20^ *BnaSROs* may also act as key functional genes in the abiotic stress response pathway, and our results provide additional evidence for the multiple stress response functions of *SROs* in plants. In addition, we further examined the expression characteristics of *BnaSROs* under ABA, MeJA and water deficit drought stress conditions using qPCR. However, although different members presented diverse response expression patterns, none of them showed regular changes. This phenomenon has been found in studies of other plant *SROs*, such as those of rice,^6^ maize,^14^ poplar,^22^ tea plant,^21^ potato^18^ and tomato,^16^ and has also been presented in the analysis of other gene families. This might be related to the different regulatory mechanisms they were subjected to during the different treatment periods. This reveals the complexity of *SROs* expression patterns while also mirroring the complexity of plant responses to stressors.

Although there were no members who were regularly expressed throughout the treatments, we screened for members who responded significantly to the different treatments. In response to ABA treatment, *BnaSRO1*, *BnaSRO2*, *BnaSRO4*, *BnaSRO8*, *BnaSRO10*, *BnaSRO11*, *BnaSRO13*, *BnaSRO15*, *BnaSRO18*, and *BnaSRO19* were significantly up-regulated at 0.5 h post-treatment, while *BnaSRO7*, *BnaSRO9*, *BnaSRO12*, and *BnaSRO16* were significantly down-regulated. In tomato, *SolySRO3*, *SolySRO4*, and *SolySRO6* were significantly up-regulated after ABA treatment, whereas *SolySRO1* and *SolySRO2* were significantly down-regulated.^16^ In potato, rice and poplar, the expression of all members of the SRO family was induced upon ABA treatment.^6,18,22^ In the MeJA treatment group, *BnaSRO1*, *BnaSRO4*, *BnaSRO6*, *BnaSRO10*, *BnaSRO11*, and *BnaSRO19* were significantly up-regulated in the early treatment group, whereas *BnaSRO2*, *BnaSRO7*, *BnaSRO8*, *BnaSRO9*, *BnaSRO12*, *BnaSRO13*, *BnaSRO14*, *BnaSRO15*, and *BnaSRO16* were up-regulated in the later treatment group. In tomato, the *SolySRO1* gene was significantly up-regulated starting 6 h after MeSA treatment and consistently continued for 24 h, while *SolySRO3* and *SolySRO4* were significantly up-regulated only at 12 h^16^. Although MeJA also plays a key role in the plant drought stress response,^45^ plant responses to ABA and MeJA treatments were inconsistent or even opposite. This may be related to the inconsistency in the signaling pathways through which they regulate the plant drought stress response. For drought treatment, most studies have used PEG treatment to simulate drought.^16,18,21,22^ In this study, we used the direct drought form of the water deficit treatment to simulate real-world conditions. We found that the expression of *BnaSRO1*, *BnaSRO4*, *BnaSRO10*, *BnaSRO11*, and *BnaSRO15* peaked 3 d after drought treatment; that of *BnaSRO6*, *BnaSRO9*, *BnaSRO12*, and *BnaSRO16* peaked 5 d after drought treatment; and that of *BnaSRO13* peaked 7 d after drought treatment. Combining the results of the different treatments, we concluded that *BnaSRO1*, *BnaSRO4*, *BnaSRO10*, *BnaSRO11*, and *BnaSRO15* deserve in-depth functional studies as important members of the drought response in rapeseed. In this study, based on the results of transcriptome expression patterns and qPCR analysis, we screened *BnaSRO1* and *BnaSRO11* as potential major drought-responsive members of the *BnaSRO* family. Both of these genes may be target genes of many transcription factor genes, especially NAC transcription factors. The *NACs* regulation of *SROs* has been identified and partially validated in previous studies. For example, in poplar, a recent study identified a number of *NAC-SRO* chains, such as *ANAC002-PtSRO2f*, *ANAC017-PtSRO2e* and *ANAC087-PtSRO2e*, through co-expression network construction, predicting that these *SROs* may be induced to respond to drought stress after interacting with NAC family proteins.^22^ In sesame, *SiSROs* may also be involved in abiotic stress response through interactions with *NAC016* and *NAC017*.^19^ In rice, *OsSRO1c* is directly regulated by *SNAC1* to enhance drought and oxidative stress tolerance.^6^ In banana, *MaSRO4* directly interacts with *MaNAC6* through the PARP domain to regulate downstream signaling pathways.^20^ Notably, we found that BnaSRO1, BnaSRO11, PtSRO2e and PtSRO2f were all in the same subgroup, further suggesting that *BnaSRO1* and *BnaSRO11* may be target genes of *NACs*. This deserves a subsequent investigated in depth. Moreover, subcellular localization predictions showed that BnaSRO1 and BnaSRO11 were localized in the nucleus, suggesting that they may also regulate other drought-responsive genes.

## Conclusions

In summary, in this study, we systematically characterized *SRO* genes in six *Brassica* crops, including *Brassica napus*, *Brassica rapa*, *Brassica nigra*, *Brassica oleracea*, *Brassica juncea* and *Brassica carinata*, and identified 19, 9, 10, 10, 18, and 20 members, respectively. Based on phylogenetic analysis, these 86 members were divided into 4 subgroups, corresponding to AtRCD1, AtSRO1, AtSRO2 and AtSRO3, and AtSRO4 and AtSRO5 of *Arabidopsis*, respectively. The number of *SRO* family members underwent significant expansion in these six *Brassica* species, with segmental duplication being the main mode of expansion. With the exception of *BnaSRO8*, the remaining 18 members had collinear genes in the *Brassica juncea* genome. The promoter regions of *BnaSROs* contain a large number of ABA- and MeJA-responsive elements, as well as transcription factor-binding and stress-responsive elements. Transcriptome data analysis and qPCR detection indicated that *BnaSROs* have multiple stress-responsive expression patterns. *BnaSRO1* and *BnaSRO11* were screened as two potential major drought-responsive members. Both of them may be target genes of *NACs* involved in drought regulation. Our study laid a foundation for the functional exploration of *SRO* genes in rapeseed and provided new genetic resources for the biological breeding of drought-tolerant rapeseed.

## Supplementary Material

Supplementary_materials_R.docx

## Data Availability

All the data relevant to this study are included in the article or uploaded as Supplementary Materials.
