# Supplementary material for: Genome-wide identification of SIMILAR to RCD ONE (SRO) gene family in rapeseed (Brassica napus L.) reveals their role in drought stress response
Source: Plant Signal Behav. 2024 Jul 14;19(1):2379128. doi: 10.1080/15592324.2024.2379128 (PMC11249032; doi:10.1080/15592324.2024.2379128)
Supplement: Supplementary_materials_R.docx [file KPSB_A_2379128_SM3259.docx]

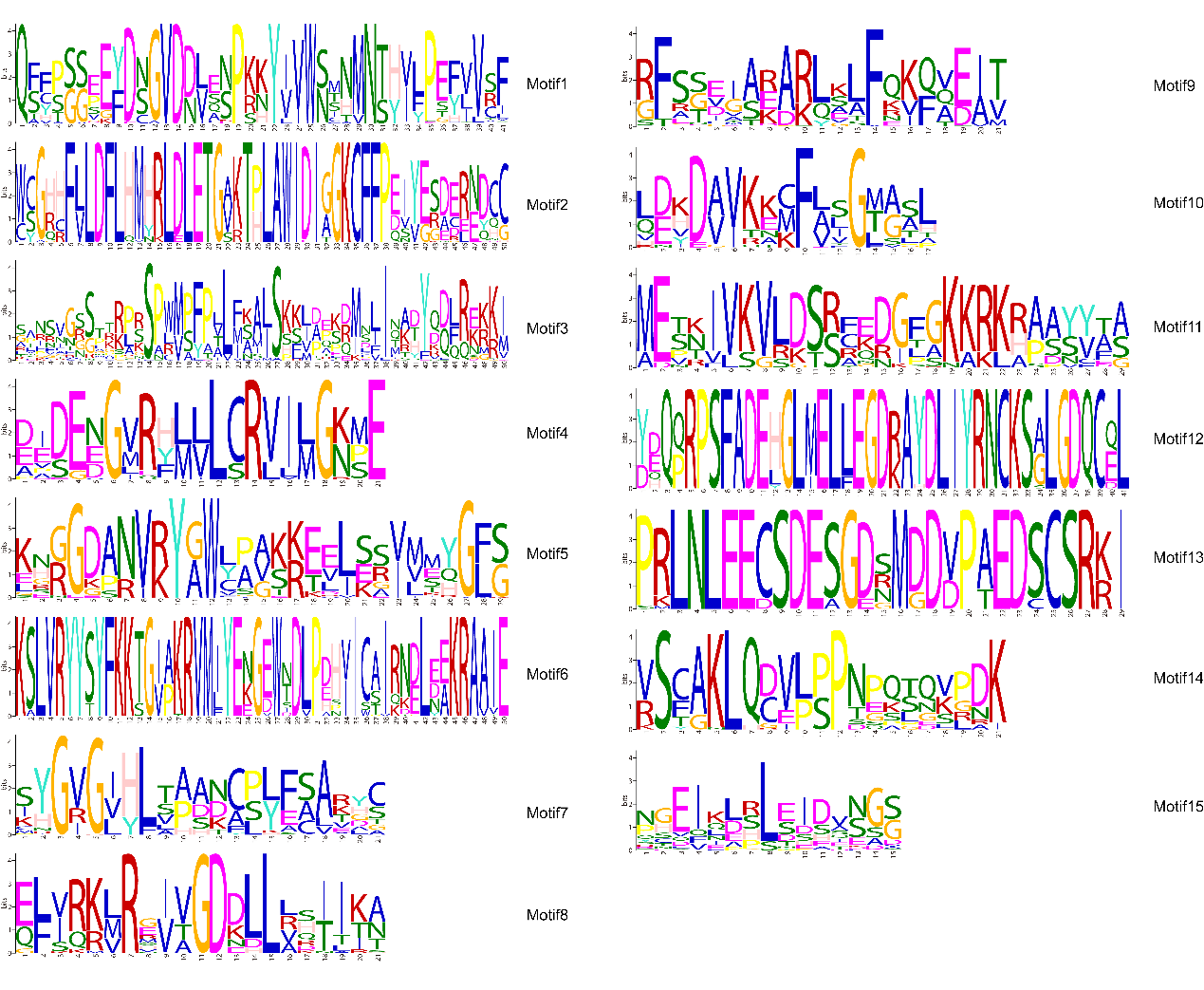


**Figure S1**. The logo map of conserved sequences of fifteen putative motifs of the SRO proteins in Figure 2.

**Table S1**. The primers used in this study.

| **Name** | **Primer sequences (5'-3')** |
| --- | --- |
| qBnaSRO1-F | TCTACCGTAACTGTAAATCTGCTC |
| qBnaSRO1-R | TGTTTCATCTCCACCGCCTC |
| qBnaSRO2-F | ATCGTCAATCTCTCTTCAAGAAAC |
| qBnaSRO2-R | CCAACACCATACTTTGACTTCTTG |
| qBnaSRO4-F | GATGCCGTTTCCTTTACTGATC |
| qBnaSRO4-R | CGTTGTATTAGTTCGCTCCTTG |
| qBnaSRO5-F | CCACGATCGTCACTATACGTAA |
| qBnaSRO5-R | CGATTCCGTTAGACGAAGAATC |
| qBnaSRO6-F | TGATGGCGGCGCAAGTGGAG |
| qBnaSRO6-R | GAGGTTTTACGCACGGAGACGA |
| qBnaSRO7-F | TTCAGGAGCTCAAAATCTCTTGT |
| qBnaSRO7-R | ACATATTCCGGGAATACATGAGT |
| qBnaSRO8-F | ATGCTGAAGGTGATATGGTTG |
| qBnaSRO8-R | AGATTTGGGTTTCGTAGTGC |
| qBnaSRO9-F | CCGATGCTGATTCCTCCTC |
| qBnaSRO9-R | CCGCGACCTCTTCCGTAGA |
| qBnaSRO10-F | TTACCAATGGATTACTCAGAGCAAG |
| qBnaSRO10-R | CCCGAGAGCAGATTTACAGTTTCG |
| qBnaSRO11-F | GTTTAGATTCGGAGCAAAAAGGA |
| qBnaSRO11-R | CGAAACCCGTTTCTGAGGATGG |
| qBnaSRO12-F | CCGCTGTTTTGTTCTTGATT |
| qBnaSRO12-R | TCTTCCTCACGGTCCCTTTC |
| qBnaSRO13-F | TGCTCTACGAGAACGGCGAATG |
| qBnaSRO13-R | AACGAAATGGCGACCGGAAC |
| qBnaSRO14-F | ATGCCGATTCCTCCTCCGGAT |
| qBnaSRO14-R | TCCGAAACGCTACGTACCTGGC |
| qBnaSRO15-F | TGCTTACGAGAATCTATCTGGG |
| qBnaSRO15-R | GAAAATGGTGACCACACCACTC |
| qBnaSRO16-F | CCACAGTTGGTGTCACACGGA |
| qBnaSRO16-R | CACAAACTCTGCTCGCGTCATC |
| qBnaSRO18-F | CCGACGACGCCTCCTCCTC |
| qBnaSRO18-R | CTCTCGTGGTGATCGCGTT |
| qBnaSRO19-F | TATAGCCGTGCTGGATGTGG |
| qBnaSRO19-R | GCAGGAAGCCATGCGTATCT |

**Table S2**. List of the *SRO* genes identified in the six *Brassica* genomes.

| Species | Genome type | Gene ID | Gene name | Chr | Start | End | AA No. | MW (kDa) | pI | GRAVY | Subcellular localization |
| --- | --- | --- | --- | --- | --- | --- | --- | --- | --- | --- | --- |
| Brassica rapa ssp.pekinensis | AA | BraA02g045920.4C.1 | *BraSRO1* | A02 | 36300646 | 36302737 | 304 | 34.15 | 8.68 | -0.38 | nucl |
|  | AA | BraA04g027400.4C.1 | *BraSRO2* | A04 | 25363637 | 25367193 | 531 | 58.63 | 6.99 | -0.41 | nucl |
|  | AA | BraA05g009970.4C.1 | *BraSRO3* | A05 | 5260895 | 5264436 | 506 | 56.47 | 6.49 | -0.39 | chlo |
|  | AA | BraA06g028360.4C.4 | *BraSRO4* | A06 | 40773918 | 40775417 | 332 | 36.90 | 8.94 | -0.32 | chlo |
|  | AA | BraA07g014760.4C.1 | *BraSRO5* | A07 | 22231334 | 22232488 | 307 | 34.04 | 8.97 | -0.32 | cyto |
|  | AA | BraA07g036480.4C.1 | *BraSRO6* | A07 | 34423095 | 34424261 | 305 | 33.79 | 8.15 | -0.32 | cyto |
|  | AA | BraA08g009560.4C.1 | *BraSRO7* | A08 | 15832545 | 15835882 | 564 | 63.20 | 6.35 | -0.45 | cyto |
|  | AA | BraA09g035450.4C.4 | *BraSRO8* | A09 | 53489205 | 53492969 | 570 | 63.51 | 5.95 | -0.49 | cyto |
|  | AA | BraA09g042530.4C.1 | *BraSRO9* | A09 | 58977114 | 58978970 | 309 | 33.42 | 6.19 | -0.22 | cyto |
| Brassica nigra | BB | BniB01g009050.2N.1 | *BniSRO1* | B01 | 4663179 | 4665323 | 556 | 62.08 | 8.35 | -0.40 | nucl |
|  | BB | BniB03g030980.2N.1 | *BniSRO2* | B03 | 14493349 | 14494510 | 311 | 34.40 | 7.75 | -0.32 | pero |
|  | BB | BniB03g035430.2N.1 | *BniSRO3* | B03 | 17348603 | 17351059 | 581 | 64.15 | 6.28 | -0.42 | nucl |
|  | BB | BniB03g048070.2N.1 | *BniSRO4* | B03 | 25307437 | 25308608 | 306 | 34.07 | 8.66 | -0.31 | cyto |
|  | BB | BniB04g002200.2N.1 | *BniSRO5* | B04 | 1057381 | 1058656 | 315 | 35.40 | 7.64 | -0.46 | nucl |
|  | BB | BniB04g038930.2N.1 | *BniSRO6* | B04 | 20931697 | 20932885 | 317 | 34.79 | 9.33 | -0.37 | pero |
|  | BB | BniB04g048420.2N.1 | *BniSRO7* | B04 | 29406084 | 29408489 | 564 | 62.60 | 5.86 | -0.46 | cyto |
|  | BB | BniB06g010790.2N.1 | *BniSRO8* | B06 | 5495096 | 5496327 | 318 | 35.63 | 8.74 | -0.44 | nucl |
|  | BB | BniB07g017280.2N.1 | *BniSRO9* | B07 | 29853627 | 29855975 | 560 | 62.47 | 5.92 | -0.50 | cyto |
|  | BB | BniB07g045100.2N.1 | *BniSRO10* | B07 | 49121045 | 49122315 | 314 | 35.22 | 8.05 | -0.35 | nucl |
| Brassica oleracea var.italica | CC | BolC2t12284H | *BolSRO1* | C02 | 21857140 | 21858358 | 357 | 40.05 | 9.27 | -0.31 | chlo |
|  | CC | BolC3t18685H | *BolSRO2* | C03 | 12268694 | 12271143 | 312 | 35.02 | 8.82 | -0.42 | nucl |
|  | CC | BolC4t22915H | *BolSRO3* | C04 | 15423601 | 15424750 | 501 | 55.94 | 7.94 | -0.43 | chlo |
|  | CC | BolC4t27920H | *BolSRO4* | C04 | 20761160 | 20762336 | 542 | 59.88 | 6.82 | -0.38 | nucl |
|  | CC | BolC5t30987H | *BolSRO5* | C05 | 29439173 | 29441521 | 305 | 33.26 | 6.32 | -0.27 | cyto |
|  | CC | BolC5t31889H | *BolSRO6* | C05 | 49680543 | 49682731 | 549 | 61.44 | 6.17 | -0.47 | cyto |
|  | CC | BolC5t32647H | *BolSRO7* | C05 | 1173229 | 1174502 | 584 | 64.39 | 6.74 | -0.43 | cyto_nucl |
|  | CC | BolC6t39500H | *BolSRO8* | C06 | 18349813 | 18351016 | 303 | 33.50 | 8.66 | -0.26 | cyto |
|  | CC | BolC7t42560H | *BolSRO9* | C07 | 25310856 | 25313261 | 313 | 34.78 | 8.33 | -0.36 | cyto |
|  | CC | BolC8t47997H | *BolSRO10* | C08 | 32743852 | 32745002 | 449 | 50.16 | 5.70 | -0.34 | cyto |
| Brassica juncea var.varuna | AABB | BjuOA02G47950 | *BjuSRO1* | A02 | 30757609 | 30759536 | 304 | 34.16 | 8.68 | -0.41 | nucl |
|  | AABB | BjuOA04G28440 | *BjuSRO2* | A04 | 18313712 | 18317609 | 531 | 58.62 | 6.99 | -0.41 | nucl |
|  | AABB | BjuOA05G09730 | *BjuSRO3* | A05 | 4987751 | 4991177 | 524 | 58.55 | 6.84 | -0.33 | chlo |
|  | AABB | BjuOA06G25830 | *BjuSRO4* | A06 | 20460029 | 20461624 | 311 | 34.84 | 8.59 | -0.41 | nucl |
|  | AABB | BjuOA07G14060 | *BjuSRO5* | A07 | 12191965 | 12193465 | 314 | 34.94 | 8.32 | -0.37 | nucl |
|  | AABB | BjuOA07G36960 | *BjuSRO6* | A07 | 24483668 | 24484839 | 307 | 34.02 | 8.44 | -0.31 | cyto |
|  | AABB | BjuOA08G09930 | *BjuSRO7* | A08 | 9143058 | 9146191 | 564 | 63.14 | 6.16 | -0.45 | cyto |
|  | AABB | BjuOA09G35240 | *BjuSRO8* | A09 | 38594748 | 38597152 | 570 | 63.41 | 5.90 | -0.50 | nucl |
|  | AABB | BjuOA09G42420 | *BjuSRO9* | A09 | 43887127 | 43888931 | 309 | 33.49 | 6.01 | -0.23 | cyto |
|  | AABB | BjuOB01G08290 | *BjuSRO10* | B01 | 4706583 | 4708727 | 541 | 60.44 | 7.56 | -0.44 | nucl |
|  | AABB | BjuOB03G29460 | *BjuSRO11* | B03 | 14712967 | 14714773 | 315 | 34.72 | 7.10 | -0.33 | pero |
|  | AABB | BjuOB03G33520 | *BjuSRO12* | B03 | 17587267 | 17591356 | 581 | 64.15 | 6.28 | -0.43 | nucl |
|  | AABB | BjuOB03G45240 | *BjuSRO13* | B03 | 25820511 | 25822077 | 307 | 34.30 | 8.80 | -0.36 | nucl |
|  | AABB | BjuOB04G02130 | *BjuSRO14* | B04 | 1073254 | 1074999 | 316 | 35.52 | 8.14 | -0.49 | nucl |
|  | AABB | BjuOB04G36450 | *BjuSRO15* | B04 | 21022141 | 21024010 | 317 | 34.84 | 9.21 | -0.40 | pero |
|  | AABB | BjuOB04G44270 | *BjuSRO16* | B04 | 29018639 | 29022366 | 556 | 61.84 | 5.89 | -0.44 | cyto |
|  | AABB | BjuOB06G11180 | *BjuSRO17* | B06 | 6169965 | 6171184 | 319 | 35.75 | 8.76 | -0.45 | nucl |
|  | AABB | BjuOB07G39550 | *BjuSRO18* | B07 | 28545802 | 28549237 | 560 | 62.47 | 6.07 | -0.50 | cyto |
| Brassica napus ssp.oleifera | AACC | BnaA02T0401000ZS | *BnaSRO1* | A02 | 34793787 | 34795093 | 303 | 34.18 | 8.68 | -0.41 | nucl |
|  | AACC | BnaA04T0226200ZS | *BnaSRO2* | A04 | 21582051 | 21584086 | 530 | 58.62 | 6.99 | -0.41 | nucl |
|  | AACC | BnaA05T0091500ZS | *BnaSRO3* | A05 | 5359864 | 5364099 | 538 | 60.27 | 7.20 | -0.35 | chlo |
|  | AACC | BnaA06T0278600ZS | *BnaSRO4* | A06 | 37903718 | 37904884 | 313 | 34.88 | 8.59 | -0.39 | nucl |
|  | AACC | BnaA07T0116500ZS | *BnaSRO5* | A07 | 15984065 | 15985227 | 307 | 34.18 | 8.75 | -0.28 | cyto |
|  | AACC | BnaA07T0318000ZS | *BnaSRO6* | A07 | 28472192 | 28473363 | 306 | 34.05 | 8.44 | -0.29 | cyto |
|  | AACC | BnaA08T0075500ZS | *BnaSRO7* | A08 | 13823018 | 13825334 | 555 | 61.90 | 6.59 | -0.41 | nucl |
|  | AACC | BnaA09T0392800ZS | *BnaSRO8* | A09 | 45290868 | 45293276 | 565 | 62.94 | 5.99 | -0.50 | cyto |
|  | AACC | BnaA09T0450900ZS | *BnaSRO9* | A09 | 50576436 | 50577597 | 308 | 33.50 | 6.01 | -0.23 | cyto |
|  | AACC | BnaC02T0533000ZS | *BnaSRO10* | C02 | 63380643 | 63382108 | 356 | 40.05 | 9.27 | -0.31 | chlo |
|  | AACC | BnaC03T0552700ZS | *BnaSRO11* | C03 | 41936412 | 41937562 | 311 | 35.02 | 8.82 | -0.42 | nucl |
|  | AACC | BnaC04T0109800ZS | *BnaSRO12* | C04 | 9972139 | 9974289 | 509 | 56.88 | 8.18 | -0.39 | chlo |
|  | AACC | BnaC04T0540200ZS | *BnaSRO13* | C04 | 66034276 | 66036415 | 537 | 59.56 | 6.66 | -0.41 | nucl |
|  | AACC | BnaC05T0207700ZS | *BnaSRO14* | C05 | 14960501 | 14961649 | 304 | 33.25 | 6.32 | -0.28 | cyto |
|  | AACC | BnaC05T0282300ZS | *BnaSRO15* | C05 | 24164501 | 24166920 | 562 | 62.88 | 6.43 | -0.49 | cyto |
|  | AACC | BnaC05T0343300ZS | *BnaSRO16* | C05 | 37874200 | 37876638 | 579 | 64.00 | 6.74 | -0.45 | cyto |
|  | AACC | BnaC06T0369900ZS | *BnaSRO17* | C06 | 47024864 | 47026064 | 305 | 33.82 | 8.81 | -0.29 | cyto |
|  | AACC | BnaC07T0173200ZS | *BnaSRO18* | C07 | 30170585 | 30171742 | 307 | 34.25 | 6.93 | -0.34 | nucl |
|  | AACC | BnaC08T0107100ZS | *BnaSRO19* | C08 | 19872936 | 19874121 | 370 | 41.99 | 5.12 | -0.30 | cyto |
| Brassica carinata | BBCC | BcaB02g09654 | *BcaSRO1* | B02 | 30170585 | 30171742 | 318 | 35.68 | 8.60 | -0.46 | nucl |
|  | BBCC | BcaB03g13750 | *BcaSRO2* | B03 | 19872169 | 19875117 | 580 | 63.99 | 6.42 | -0.42 | nucl |
|  | BBCC | BcaB03g14144 | *BcaSRO3* | B03 | 30757609 | 30759536 | 308 | 34.28 | 8.69 | -0.33 | pero |
|  | BBCC | BcaB03g14803 | *BcaSRO4* | B03 | 18313712 | 18317609 | 300 | 33.53 | 8.45 | -0.39 | nucl |
|  | BBCC | BcaB04g17989 | *BcaSRO5* | B04 | 4987751 | 4991177 | 559 | 62.47 | 5.84 | -0.50 | cyto |
|  | BBCC | BcaB06g28846 | *BcaSRO6* | B06 | 20460029 | 20461624 | 543 | 60.48 | 7.21 | -0.43 | nucl |
|  | BBCC | BcaB07g29844 | *BcaSRO7* | B07 | 12191965 | 12193465 | 314 | 35.41 | 7.64 | -0.47 | nucl |
|  | BBCC | BcaB07g32345 | *BcaSRO8* | B07 | 24483668 | 24484839 | 316 | 34.61 | 5.73 | -0.47 | nucl |
|  | BBCC | BcaB07g32898 | *BcaSRO9* | B07 | 9143058 | 9146191 | 563 | 62.60 | 5.86 | -0.46 | cyto |
|  | BBCC | BcaC01g02483 | *BcaSRO10* | C01 | 38594748 | 38597152 | 311 | 35.02 | 8.82 | -0.42 | nucl |
|  | BBCC | BcaC02g07707 | *BcaSRO11* | C02 | 43887127 | 43888931 | 537 | 59.56 | 6.66 | -0.41 | nucl |
|  | BBCC | BcaC02g11656 | *BcaSRO12* | C03 | 4706583 | 4708727 | 495 | 55.35 | 6.95 | -0.45 | chlo |
|  | BBCC | BcaC03g18157 | *BcaSRO13* | C03 | 14712967 | 14714773 | 303 | 34.08 | 8.58 | -0.35 | cyto |
|  | BBCC | BcaC03g18162 | *BcaSRO14* | C03 | 17587267 | 17591356 | 291 | 32.77 | 7.61 | -0.38 | cyto |
|  | BBCC | BcaC05g26277 | *BcaSRO15* | C05 | 25820511 | 25822077 | 291 | 31.94 | 6.00 | -0.36 | nucl |
|  | BBCC | BcaC05g27256 | *BcaSRO16* | C05 | 1073254 | 1074999 | 562 | 62.86 | 6.31 | -0.50 | cyto |
|  | BBCC | BcaC05g27903 | *BcaSRO17* | C05 | 21022141 | 21024010 | 579 | 64.00 | 6.74 | -0.45 | cyto |
|  | BBCC | BcaC06g35043 | *BcaSRO18* | C06 | 29018639 | 29022366 | 308 | 34.27 | 8.42 | -0.31 | cyto |
|  | BBCC | BcaC07g37627 | *BcaSRO19* | C07 | 6169965 | 6171184 | 495 | 56.14 | 7.55 | -0.44 | cyto |
|  | BBCC | BcaC08g46487 | *BcaSRO20* | C08 | 28545802 | 28549237 | 300 | 33.21 | 8.66 | -0.30 | cyto |

**Table S3.** The accession numbers of the SRO proteins in Figure 1.

| **Gene name** | **Accession number** | **Species** |
| --- | --- | --- |
| AtRCD1 | AT1G32230.1 | Arabidopsis thaliana |
| AtSRO1 | AT2G35510.1 |  |
| AtSRO2 | AT1G23550.1 |  |
| AtSRO3 | AT1G70440.1 |  |
| AtSRO4 | AT3G47720.1 |  |
| AtSRO5 | AT5G62520.1 |  |
| OsSRO1a | Os10g42710 | Oryza sativa |
| OsSRO1b | Os03g63770 |  |
| OsSRO1c | Os03g12820 |  |
| OsSRO1d | Os06g13860 |  |
| OsSRO1e | Os04g57640 |  |
| ZmSRO1 | GRMZM2G122543_P01 | Zea mays |
| ZmSRO2 | GRMZM5G866843_P03 |  |
| ZmSRO3 | GRMZM2G011469_P01 |  |
| ZmSRO4 | GRMZM2G072894_P01 |  |
| ZmSRO5 | GRMZM2G145236_P02 |  |
| ZmSRO6 | GRMZM2G177878_P02 |  |
| GmSRO1a | Glyma09g34000 | Glycine max |
| GmSRO1b | Glyma01g01900 |  |
| GmSRO2a | Glyma08g12963 |  |
| GmSRO2b | Glyma05g02210 |  |
| GmSRO2c | Glyma17g09720 |  |
| GmSRO2d | Glyma04g35560 |  |
| TaSRO1b_1_4A | TraesCS4A02G059400.1 | Triticum aestivum |
| TaSRO1b_1_4B | TraesCS4B02G150300.1 |  |
| TaSRO1b_1_4D | TraesCS4D02G236400.1 |  |
| TaSRO2a_1_1A | TraesCS1A02G195400.1 |  |
| TaSRO2a_1_1B | TraesCS1B02G210300.1 |  |
| TaSRO2a_1_1D | TraesCS1D02G199200.1 |  |
| TaSRO2a_2_4A | TraesCS4A02G321300.1 |  |
| TaSRO2a_3_5B | TraesCS5B02G557500.1 |  |
| TaSRO2a_3_5D | TraesCS5D02G551800.1 |  |
| TaSRO2a_4_7B | TraesCS7B02G105400.1 |  |
| TaSRO2a_4_7D | TraesCS7D02G201500.1 |  |
| TaSRO2a_5_7A | TraesCS7A02G409200.1 |  |
| TaSRO2a_5_7B | TraesCS7B02G309100.1 |  |
| TaSRO2a_5_7D | TraesCS7D02G402500.1 |  |
| TaSRO2b_1_1A | TraesCS1A02G038400.1 |  |
| TaSRO2b_1_1B | TraesCS1B02G049300.1 |  |
| TaSRO2b_1_1D | TraesCS1D02G040100.1 |  |
| TaSRO2b_2_1A | TraesCS1A02G328000.1 |  |
| TaSRO2b_2_1B | TraesCS1B02G341700.1 |  |
| TaSRO2b_2_1D | TraesCS1D02G330500.1 |  |
| TaSRO2b_3_4A | TraesCS4A02G163100.1 |  |
| TaSRO2b_3_4B | TraesCS4B02G235000.1 |  |
| TaSRO2b_3_4D | TraesCS4D02G160000.1 |  |
| TaSRO2b_4_6A | TraesCS6A02G242900.1 |  |
| TaSRO2b_4_6B | TraesCS6B02G281300.1 |  |
| TaSRO2b_4_6D | TraesCS6D02G225200.1 |  |
| TaSRO2b_5_6B | TraesCS6B02G410300.1 |  |
| TaSRO2b_6_7A | TraesCS7A02G414700.1 |  |
| TaSRO2b_6_7B | TraesCS7B02G314600.1 |  |
| TaSRO2b_6_7D | TraesCS7D02G407800.1 |  |
| PtSRO2d | Potri.006G231500 | Populus trichocarpa |
| PtSRO2e | Potri.012G081100 |  |
| PtSRO2c | Potri.006G231100 |  |
| PtSRO2f | Potri.015G076500 |  |
| PtSRO2a | Potri.018G055100 |  |
| PtSRO2b | Potri.006G231600 |  |
| PtSRO1c | Potri.002G112300 |  |
| PtSRO1b | Potri.001G137200 |  |
| PtSRO1a | Potri.003G096700 |  |

**Table S4**. The Ka/Ks ratios of co-linear SRO genes between Brassica napus and Brassica juncea genomes.

| **Seq_1** | **Seq_2** | **Ka** | **Ks** | **Ka/Ks** |
| --- | --- | --- | --- | --- |
| BnaSRO1 | BjuSRO1 | 0.0014 | 0.0048 | 0.2976 |
| BnaSRO1 | BjuSRO4 | 0.0817 | 0.4311 | 0.1895 |
| BnaSRO1 | BjuSRO14 | 0.0373 | 0.2113 | 0.1768 |
| BnaSRO1 | BjuSRO17 | 0.0785 | 0.3958 | 0.1983 |
| BnaSRO2 | BjuSRO2 | 0 | 0 | - |
| BnaSRO2 | BjuSRO3 | 0.1338 | 0.3439 | 0.3892 |
| BnaSRO2 | BjuSRO10 | 0.067 | 0.1891 | 0.3541 |
| BnaSRO3 | BjuSRO2 | 0.1533 | 0.3811 | 0.4022 |
| BnaSRO3 | BjuSRO3 | 0.049 | 0.0952 | 0.5142 |
| BnaSRO3 | BjuSRO10 | 0.1446 | 0.3542 | 0.4083 |
| BnaSRO4 | BjuSRO1 | 0.0835 | 0.3825 | 0.2184 |
| BnaSRO4 | BjuSRO4 | 0.0056 | 0.0379 | 0.1484 |
| BnaSRO4 | BjuSRO14 | 0.0894 | 0.4604 | 0.1942 |
| BnaSRO4 | BjuSRO17 | 0.0363 | 0.281 | 0.1293 |
| BnaSRO5 | BjuSRO5 | 0.015 | 0.0599 | 0.2506 |
| BnaSRO5 | BjuSRO9 | 0.1793 | 0.5054 | 0.3546 |
| BnaSRO5 | BjuSRO11 | 0.0759 | 0.2089 | 0.3631 |
| BnaSRO5 | BjuSRO15 | 0.1387 | 0.4577 | 0.3031 |
| BnaSRO6 | BjuSRO6 | 0.0071 | 0.0335 | 0.2128 |
| BnaSRO6 | BjuSRO9 | 0.2801 | 1.1069 | 0.2531 |
| BnaSRO6 | BjuSRO13 | 0.0578 | 0.2264 | 0.2554 |
| BnaSRO7 | BjuSRO7 | 0.0306 | 0.0365 | 0.8389 |
| BnaSRO7 | BjuSRO8 | 0.1124 | 0.3795 | 0.2963 |
| BnaSRO7 | BjuSRO12 | 0.1243 | 0.311 | 0.3999 |
| BnaSRO7 | BjuSRO16 | 0.114 | 0.391 | 0.2916 |
| BnaSRO7 | BjuSRO18 | 0.0802 | 0.1774 | 0.452 |
| BnaSRO9 | BjuSRO5 | 0.1801 | 0.5215 | 0.3453 |
| BnaSRO9 | BjuSRO6 | 0.2786 | 1.1408 | 0.2442 |
| BnaSRO9 | BjuSRO9 | 0.0014 | 0.0184 | 0.0774 |
| BnaSRO9 | BjuSRO13 | 0.2724 | 1.4329 | 0.1901 |
| BnaSRO9 | BjuSRO11 | 0.1792 | 0.4592 | 0.3903 |
| BnaSRO9 | BjuSRO15 | 0.1109 | 0.2677 | 0.4145 |
| BnaSRO10 | BjuSRO1 | 0.0189 | 0.0965 | 0.1961 |
| BnaSRO10 | BjuSRO4 | 0.0771 | 0.4606 | 0.1674 |
| BnaSRO10 | BjuSRO14 | 0.03 | 0.2493 | 0.1205 |
| BnaSRO10 | BjuSRO17 | 0.0734 | 0.4299 | 0.1708 |
| BnaSRO11 | BjuSRO1 | 0.0861 | 0.3746 | 0.2299 |
| BnaSRO11 | BjuSRO4 | 0.0134 | 0.1123 | 0.1194 |
| BnaSRO11 | BjuSRO14 | 0.096 | 0.4593 | 0.209 |
| BnaSRO11 | BjuSRO17 | 0.0357 | 0.2496 | 0.1432 |
| BnaSRO12 | BjuSRO2 | 0.1313 | 0.3188 | 0.4117 |
| BnaSRO12 | BjuSRO3 | 0.0469 | 0.1036 | 0.4531 |
| BnaSRO12 | BjuSRO10 | 0.1183 | 0.3102 | 0.3814 |
| BnaSRO13 | BjuSRO2 | 0.0244 | 0.0773 | 0.3158 |
| BnaSRO13 | BjuSRO3 | 0.1262 | 0.3328 | 0.3791 |
| BnaSRO13 | BjuSRO10 | 0.0634 | 0.1826 | 0.3474 |
| BnaSRO14 | BjuSRO5 | 0.1456 | 0.4197 | 0.3469 |
| BnaSRO14 | BjuSRO6 | 0.2666 | 1.3062 | 0.2041 |
| BnaSRO14 | BjuSRO9 | 0.0661 | 0.1374 | 0.481 |
| BnaSRO14 | BjuSRO11 | 0.1565 | 0.4016 | 0.3895 |
| BnaSRO14 | BjuSRO13 | 0.2458 | 1.4542 | 0.169 |
| BnaSRO14 | BjuSRO15 | 0.0654 | 0.2049 | 0.3194 |
| BnaSRO16 | BjuSRO7 | 0.1035 | 0.311 | 0.3329 |
| BnaSRO16 | BjuSRO8 | 0.0891 | 0.3505 | 0.2542 |
| BnaSRO16 | BjuSRO12 | 0.043 | 0.1233 | 0.3486 |
| BnaSRO16 | BjuSRO16 | 0.0868 | 0.3791 | 0.2288 |
| BnaSRO16 | BjuSRO18 | 0.1025 | 0.3369 | 0.3042 |
| BnaSRO17 | BjuSRO6 | 0.0301 | 0.0702 | 0.4282 |
| BnaSRO17 | BjuSRO5 | 0.2868 | 0.9378 | 0.3058 |
| BnaSRO17 | BjuSRO13 | 0.0633 | 0.2177 | 0.2905 |
| BnaSRO17 | BjuSRO11 | 0.2859 | 0.991 | 0.2885 |
| BnaSRO17 | BjuSRO15 | 0.2636 | 1.0399 | 0.2535 |
| BnaSRO18 | BjuSRO5 | 0.0332 | 0.0885 | 0.3751 |
| BnaSRO18 | BjuSRO6 | 0.2776 | 0.9893 | 0.2806 |
| BnaSRO18 | BjuSRO9 | 0.1761 | 0.5193 | 0.3392 |
| BnaSRO18 | BjuSRO11 | 0.0744 | 0.1866 | 0.3986 |
| BnaSRO18 | BjuSRO15 | 0.1279 | 0.47 | 0.2721 |
| BnaSRO19 | BjuSRO7 | 0.0465 | 0.0699 | 0.6643 |
| BnaSRO19 | BjuSRO8 | 0.086 | 0.4107 | 0.2095 |
| BnaSRO19 | BjuSRO12 | 0.0821 | 0.3096 | 0.265 |
| BnaSRO19 | BjuSRO16 | 0.0827 | 0.3519 | 0.235 |
| BnaSRO19 | BjuSRO18 | 0.0819 | 0.2109 | 0.3884 |

**Table S5**. Arabidopsis homologous genes corresponding to transcription factor genes in rapeseed shown in Figure 8. Members that may regulate both BnaSRO1 and BnaSRO11 are shown in bold font.

| **TF Gene ID** | **At Gene ID** | **At Name** |
| --- | --- | --- |
| BnaA01G0024300ZS | AT4G36160 | NAC076 |
| BnaA01G0357400ZS | AT3G13810 | IDD11 |
| BnaA01G0404700ZS | AT3G04060 | NAC046 |
| BnaA02G0007900ZS | AT5G02460 | DOF5.1 |
| BnaA02G0069800ZS | AT5G17430 | BBM |
| **BnaA02G0136000ZS** | AT5G53950 | NAC098 |
| BnaA02G0320500ZS | AT2G02070 | IDD5 |
| BnaA03G0444300ZS | AT4G17980 | NAC071 |
| BnaA04G0186400ZS | AT2G28810 | DOF2.2 |
| **BnaA05G0382300ZS** | AT3G17730 | NAC057 |
| BnaA06G0016100ZS | AT3G15510 | NAC2 |
| BnaA06G0020300ZS | AT1G52150 | HB-15 |
| BnaA06G0297600ZS | AT5G64750 | ABR1 |
| BnaA07G0144100ZS | AT5G66940 | DOF5.8 |
| BnaA07G0154400ZS | AT2G27300 | NTL8 |
| **BnaA07G0170900ZS** | AT5G39610 | NAC2 |
| **BnaA07G0360800ZS** | AT1G76420 | NAC031 |
| BnaA08G0020700ZS | AT1G51700 | DOF1.7 |
| BnaA08G0032200ZS | AT1G49480 | REM19 |
| BnaA09G0089600ZS | AT5G64750 | ABR1 |
| BnaA09G0098700ZS | AT5G66730 | IDD1 |
| BnaA09G0409100ZS | AT1G30490 | HB-9 |
| BnaA09G0686600ZS | AT1G03840 | MGP |
| BnaC01G0012600ZS | AT4G38000 | DOF4.7 |
| BnaC01G0028400ZS | AT4G36160 | NAC076 |
| **BnaC01G0267100ZS** | AT3G50410 | DOF3.4 |
| **BnaC01G0434000ZS** | AT3G15170 | NAC054 |
| BnaC01G0454000ZS | AT3G12910 | NAC |
| **BnaC01G0505500ZS** | AT3G03200 | NAC045 |
| BnaC02G0009200ZS | AT5G03150 | JKD |
| **BnaC02G0409500ZS** | AT5G46590 | NAC096 |
| BnaC03G0100100ZS | AT5G20240 | PI |
| **BnaC03G0509300ZS** | AT2G18060 | NAC037 |
| BnaC04G0081800ZS | AT2G38340 | DREB2E |
| BnaC04G0457400ZS | AT2G24430 | NAC039 |
| **BnaC05G0330800ZS** | AT1G34180 | NAC016 |
| BnaC05G0416100ZS | AT3G18400 | NAC058 |
| BnaC05G0555600ZS | AT3G04060 | NAC046 |
| **BnaC06G0162600ZS** | AT5G39820 | NAC094 |
| **BnaC07G0178000ZS** | AT1G32870 | NAC13 |
| **BnaC07G0215900ZS** | AT5G66300 | NAC105 |
| BnaC07G0269400ZS | AT2G01570 | RGA |
| BnaC07G0275700ZS | AT2G02080 | IDD4 |
| BnaC07G0547100ZS | AT4G38910 | BPC5 |
| **BnaC08G0250500ZS** | AT1G19040 | NAC |
